# Revisiting novel word semantic priming: The role of strategic priming mechanisms

**DOI:** 10.1177/17470218241306747

**Published:** 2024-12-21

**Authors:** Lewis V Ball, Perrine Brusini, Colin Bannard

**Affiliations:** 1 Department of Psychology, University of Liverpool, Liverpool, UK; 2 Department of Psychology, University of York, York, UK; 3 Department of Linguistics and English Language, University of Manchester, Manchester, UK

**Keywords:** Lexical representation, word learning, complimentary learning systems, semantic priming, strategic processing

## Abstract

Although it has been proposed that new words are encoded in a qualitatively different way from established words—in episodic rather than semantic memory—such accounts are challenged by the finding that newly learnt words influence the processing of well-known words in semantic priming tasks. In this article, we explore whether this apparent contradiction is due to differences in task design. Specifically, we hypothesised that a large stimulus onset asynchrony (SOA) would allow the participant to engage strategic retrieval and priming mechanisms to facilitate the recognition of a semantically related word, compared with a shorter SOA, which promotes more automatic processing. In Experiment 1, 60 participants learned 34 novel words and their meanings that later served as primes for related/unrelated existing word targets in a primed lexical decision task, with a 450 ms SOA. There was no significant priming effect. In Experiment 2, we increased the SOA to 1,000 ms, and found a significant priming effect with novel words. Finally, there was no significant priming effect with novel words in Experiment 3 that used a 200 ms SOA. A semantic priming effect with familiar words was found in Experiments 1 and 3, but not Experiment 2 (the longest SOA). We interpret these results as providing evidence for the idea that new and existing words are represented differently, with the former encoded outside of conventional language networks as they appear to rely predominantly on slow (strategic) mechanisms to prime related, existing words.

## Introduction

It is estimated that, on average, an adult speaker of American English understands around 42,000 base word forms (Brysbaert et al., 2016). Although our lexicons experience a greater influx of new vocabulary during childhood compared with adulthood, word learning continues throughout one’s lifetime (Ramscar et al., 2014), such as when we are presented with new word forms and meanings (e.g., podcast, broadband). We also continue to update our understanding of known words (Klooster & Duff, 2015; Klooster et al., 2020). Understanding how we convert transient encounters with words into long-term semantic knowledge is thus one of the core questions in psycholinguistic research.

One popular account of this process calls on the Complementary Learning Systems (CLS) account of memory (McClelland, 2013; McClelland et al., 1995). This account proposes that lexical acquisition follows a two-stage learning process (Davis & Gaskell, 2009; Lindsay & Gaskell, 2010). The first stage involves the rapid acquisition of new words in episodic memory that is supported by cortico-hippocampal representations. At this stage of learning, the hippocampus mediates the mapping between relevant cortical language areas, such as the mapping between areas of the cortex involved in word-form and meaning representations, respectively. The second stage involves the integration of this knowledge into core language networks, and the formation of direct cortical mappings (i.e., direct cortical links between word-form and meaning representations). Such integration is thought to be facilitated by offline consolidation periods, such as sleep (Palma & Titone, 2021; Schimke et al., 2021), which promotes hippocampal replay and reactivation, supporting the consolidation of new knowledge into cortical networks (Schapiro et al., 2018; Stickgold & Walker, 2005, 2013; Tamminen et al.,2010, 2013).

One way to probe representational differences between new and existing words is through semantic priming (Meyer & Schvaneveldt, 1971; see McNamara, 2005, for a review). When completing a primed lexical decision task (pLDT), for example, participants are known to respond more quickly to a real word target (e.g., doctor) when it is preceded by a semantically related prime (e.g., nurse) than when it is preceded by an unrelated prime word (e.g., chair). Under certain conditions, this “semantic priming effect” is thought to be caused by “spreading activation” occurring in the semantic network—when the prime word is processed, activation spreads to associated concepts, reducing their activation threshold for recognition (Collins & Loftus, 1975; Posner & Snyder, 1975). Importantly, though, such mechanisms may only occur once a word has been integrated with other concepts in the semantic network (Davis & Gaskell, 2009). Hence, according to the CLS account, semantic priming with new words is dependent on offline consolidation periods that promote the shift from episodic to cortical representation.

Supporting evidence for this claim, however, is quite mixed. Several studies have indeed reported no priming effects when recently learned words (words for which no period(s) of offline consolidation has yet taken place) are used as primes in a pLDT (Bakker-Marshall et al., 2018; Batterink & Neville, 2011; Borovsky et al., 2012; Coutanche & Thompson-Schill, 2014; Tamminen & Gaskell, 2013; van der Ven et al., 2015, 2017). For example, Tamminen and Gaskell (2013; hereafter abbreviated to TG13) taught participants 68 novel words along with their meanings (*feckton—*a type of cat that has stripes and is bluish-grey) across two training sessions—one completed at least a day before the critical testing phase (remote condition), and one completed immediately before the testing phase (recent condition). The testing phase consisted of a pLDT, where the 68 learned words acted as primes for related (e.g., dog, mouse, kitten) and unrelated familiar targets. Despite extensive training, novel words in the recent condition did not facilitate the recognition of their related targets. In further support of the CLS account, some studies have also found evidence of priming after a consolidation period had taken place (Coutanche & Thompson-Schill, 2014; van der Ven et al., 2015, 2017), including the remote condition in TG13, perhaps indicative of the (at least partial) integration of novel knowledge into cortical language networks.

On the contrary, several studies have reported significant effects soon after learning, without offline consolidation (Bakker et al., 2015; Balass et al., 2010; Perfetti et al., 2005; for a review see McMurray et al., 2016). Furthermore, studies which measured electroencephalography (EEG) during novel word processing have reported significant “N400 priming effects” (Balass et al., 2010; Borovsky et al., 2012; Mestres-Missé et al., 2007; Perfetti et al., 2005) in a semantic priming paradigm, immediately after acquisition. This is intriguing because the N400 component is argued by some researchers (Kutas & Federmeier, 2000; Lau et al., 2008) to represent the ease of accessing a word from the lexicon, and hence has been used in word learning studies as an electrophysiological marker for the integration of words into the semantic network.

In sum, the existing literature does not present an entirely conclusive picture regarding the semantic priming capabilities of new words. Resolving these apparent contradictions is vital if we are to propose coherent theories of word acquisition. In this article, we aim to reconcile existing findings by focusing on the potential influence of task design in the context of semantic priming with new words. To do so, in the following text, we outline alternative mechanisms of semantic priming to spreading activation, before speculating about the relative contributions of these mechanisms in prior work.

The spreading activation account of semantic priming considers it to be an automatic process resulting from swift and non-volitional activity in the semantic network (Hutchison, 2003). There are, however, other mechanisms that may underlie the semantic priming effect. These mechanisms are strategic and controlled—they may be explicitly recruited by the participant to facilitate their performance on the task at hand. An example of a strategic priming mechanism is the expectancy generation account (Becker, 1980; Posner & Snyder, 1975). Under this account, participants make active predictions regarding the upcoming target’s identity, based on the retrieved meaning of the prime. If the target is indeed predicted (i.e., the participant correctly predicted its presence), then its recognition is facilitated. However, recognition is inhibited when the target is not predicted.

Another type of a strategic priming mechanism is semantic matching (Neely et al., 1989; Neely & Keefe, 1989). Under this account, the participant “checks back” the meaning of the target with the retrieved meaning of the prime, searching for a relationship. In a pLDT, if a relationship is recognised, this can bias and facilitate the participant to respond with a “word” response; the target must be a real word for there to be a relationship with the prime. If, however, no relationship is detected but the target is a real word, as is the case on unrelated prime- (word)target trials, the participant must override the bias to respond “nonword,” delaying response time.

The likelihood of the participant recruiting either of these mechanisms is dependent on the stimulus onset asynchrony (SOA; McNamara, 2005), which refers to the temporal delay between the presentation of the prime and the presentation of the target. Although there is no absolute SOA threshold for determining an automatic—strategic division (Hutchison, 2003), as the SOA increases, so does the propensity for strategic mechanisms to emerge (de Groot, 1984; den Heyer et al., 1983, 1985; Favreau & Segalowitz, 1983; Neely, 1977). Hence, lower SOAs are thought to be more closely coupled with automatic processing.

In the existing literature, the following SOAs have been used to measure novel word semantic priming: 47 ms (Tamminen & Gaskell, 2013, Experiment 2), 200 ms (Coutanche & Thompson-Schill, 2014), 250 ms (van der Ven et al., 2015, 2017), 450 ms (Tamminen & Gaskell, 2013, Experiment 1), 500 ms (Bakker et al., 2015; Bakker-Marshall et al., 2018; Batterink & Neville, 2011; Borovsky et al., 2012), and 1,000 ms (Balass et al., 2010; Perfetti et al., 2005). The latter two SOAs, in particular, fall within the temporal window argued to be within the development of strategic processes (McNamara, 2005; Neely, 1977). Intriguingly, studies that have reported significant priming effects with new words (Bakker et al., 2015; Balass et al., 2010; Perfetti et al., 2005) have, therefore, used an SOA that is considered long enough to encourage the use of strategic processing. What is more, all three of these studies used a semantic judgement task to measure priming. In this task, all target words are real words, and participants are instructed to decide if the prime and target are semantically related. Thus, semantic processing can be viewed as being more explicit in a semantic judgement task, with participants required to make use of word meaning, compared with a pLDT where meaning processing is not necessary in determining the lexical status of the target word.

One possibility, then, is that novel words engage in semantic processing using strategic mechanisms. Crucially, such processing may be able to act upon episodic memory traces (Bakker et al., 2015), which are thought to regulate word knowledge in its early stages (Davis & Gaskell, 2009). If this is true, then novel words may engage in semantic priming through strategic processes, which would require a sufficiently long SOA. This is compared with the behaviour of familiar words that may also prime under more automatic conditions (i.e., a shorter SOA) due to integrated semantic representations. Such a distinction in priming according to the mechanism of action, however, has not been directly tested before.

This study investigated the mechanisms and parameters that may influence novel word semantic processes. We report three experiments in which we replicated part of the design and stimuli used in TG13. Specifically, we replicated the parameters of their recent condition. Thirty-four novel words and their meanings were taught to participants, which later served as primes in a pLDT. Thus, this study does not assess the effect of time and/or consolidation on novel word semantic priming (which was central to the TG13 study). In Experiment 1, we used an SOA of 450 ms (as per Experiment 1 in TG13) in the pLDT. Consistent with TG13, we predicted that we would observe significant semantic priming with familiar prime words but not with recently learned novel words. In Experiment 2, we increased the SOA to 1,000 ms, with all other experimental parameters kept constant relative to Experiment 1. We anticipated that the increased SOA would encourage the use of strategic mechanisms to emerge, and therefore predicted a significant semantic priming effect with both familiar and novel words in Experiment 2. Finally, in Experiment 3, we reduced the SOA to 200 ms. Relative to Experiments 1 and 2, semantic priming in Experiment 3 was anticipated to be underpinned most strongly by automatic priming mechanisms. As with Experiment 1, we predicted that we would observe a dissociation in semantic priming, with significant priming with familiar prime words only.

### Data availability

This study was pre-registered ahead of data collection on Open Science Framework (https://osf.io/v9hwx/). The data and analysis scripts for this study can be accessed here: https://osf.io/6xvzp/

## Experiment 1

### Methods

#### Participants

Participants were recruited through *Prolific*—an online platform for participant recruitment https://www.prolific.co/—and received £11 for their participation. Sixty-one participants completed the experiment in total, of which 60 contributed data to the analysis (*M* age = 40.59 years; *SD* = ±13.58; 27 males). One participant was removed from data analysis for failing to provide a single correct response in the pLDTs. All participants reported no known language-related disorders and reported themselves to be native speakers of English. As outlined in the pre-registration, we recruited the same number of participants as TG13. We aimed to achieve the same number of participants as TG13 as our experiments recruited the same stimuli and very similar procedures as TG13, which reported significant priming effects. Thus, we deemed it reasonable to expect that 60 participants would provide sufficient statistical power to observe semantic priming under these experimental parameters. Ethical approval (for all three experiments) was obtained from the University of Liverpool Health and Life Sciences Research Ethics Committee.

#### Design and stimuli

The published TG13 article is derived from work contained in a PhD thesis (Tamminen, 2010), which contains the stimuli used. When describing the method of this study, we are largely presenting information that is contained within the thesis and the related published article (Tamminen & Gaskell, 2013). There are, however, some minor differences between the methods of this study and that described in TG13, which will be highlighted.

In this study, 34 novel words (e.g., *blontack*—see Supplemental Appendix A for a full list of novel words) and their meanings (is a type of cat that has stripes and is bluish-grey) were selected. We specifically selected the 34 novel words and meanings that were used in Experiment 2 of Tamminen and Gaskell (2013). Thirty-four familiar words (e.g., clinic—see Supplemental Appendix A for a full list of familiar primes), also used in TG13, were selected and used as primes in the familiar pLDT. This familiar task was included to establish a baseline measure of semantic priming. For full details of stimuli characteristics, as well as details of the selection process for identifying familiar target words and creating non-word targets that were used in the primed lexical decisions tasks, we direct the reader to the study by Tamminen and Gaskell (2013).

#### Procedure

The experiment took place online via Gorilla Experiment Builder (Anwyl-Irvine et al., 2020—https://app.gorilla.sc). This contrasts from TG13 where data collection took place in the lab. The experiment was restricted to PC or Mac users (no tablets or other mobile devices were allowed). Participants provided informed consent before the experiment began.

**Figure 1.**
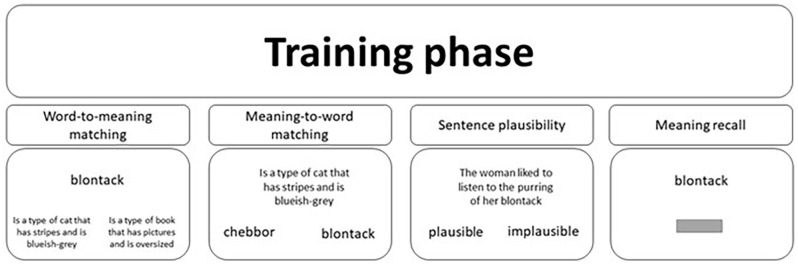
A visual depiction of the four training tasks used in this study. The grey horizontal bar in the meaning recall task represents the response box participants were provided with to type the meaning of the cued novel word.

The experiment was divided into two key sections: the training phase and testing phase. The training phase was designed to teach participants the meanings of the 34 novel words and consisted of a series of distinct tasks: a word-to-meaning matching task, a meaning-to-word matching task, a sentence plausibility task and a meaning recall task. Figure 1 depicts all four training tasks.

In the “word-to-meaning matching” task, a novel word was presented in the centre of the screen. Below this were two meanings in the left and right quadrants—one of which was the meaning of the on-screen novel word, whereas the other was the meaning of a different novel word. The participant was required to select, using their mouse cursor, the correct meaning of the on-screen word. The “meaning-to-word matching” task was very similar, except this time a meaning was displayed on-screen, and below were two novel word alternatives, with participants asked to select the word that referred to the on-screen meaning.

For both tasks, the correct response appeared an equal number of times on both sides. Across participants, the correct response was always paired with the same foil word/meaning. This appears to differ from TG13 where “ . . . the incorrect option was randomly picked from the pool of [words/]meanings used in this session by the experimental software.” (Tamminen & Gaskell, 2013, p. 1009). In both tasks, the correct word/meaning remained on-screen for 1,500 ms following the participant’s response, and unlimited time was allowed to provide a response. Within each block (of both tasks), each word/meaning was presented as a response option twice: once as the correct response and once as the incorrect foil.

In the “sentence plausibility” task, the novel words were presented at the end of a sentence. Based on the meaning of the novel word (e.g., *blontack—*is a type of cat that has stripes and is bluish-grey), participants were asked to judge whether the sentence was plausible (e.g., “The woman liked to listen to the purring of her *blontack*”) or implausible (e.g., “The monkey was too frightened to climb the *blontack*”). The sentence was presented in the centre of the screen, with the options “plausible” and “implausible” presented below in the left and right quadrants, respectively. Each novel word was presented four times throughout this task, three times within a plausible sentence and once within an implausible sentence. This imbalance was designed to minimise the novel word’s appearances in the presence of an incorrect meaning that might interfere with learning. On each presentation, a different sentence was used. Following the participant’s response, feedback was provided in the form of a green tick for a correct response and a red cross for an incorrect response. The novel word and its meaning were then presented on screen for 1,500 ms. The reader is directed to Supplemental Appendix B for a full list of training sentences.

In the “meaning recall” task, participants were presented with a novel word in the centre of the screen and were prompted to type the meaning of the on-screen word. Unlimited time was allowed, and the correct meaning was displayed on-screen for 1,500 ms following the participant’s response. Participants were encouraged to type the full meaning of the word to the best of their ability. Within a single block of the meaning recall task, each novel word was presented once.

The order of the training tasks throughout the training phase is as follows. First, participants completed three blocks of the word-to-meaning task followed by one block of the meaning recall task. This was followed by two more blocks of the word-to-meaning matching task followed by another single block of meaning recall. Following this was three blocks of the meaning-to-word matching task followed by another, and final, single block of meaning recall. Finally, two more blocks of the meaning-to-word matching task were followed by four blocks of the sentence plausibility task. Across all training tasks, the presentation of trials was randomised across participants. Participants were in control of when each training block commenced and were instructed that they could use the time between blocks to take a short break.

Following training participants immediately moved onto the “testing phase,” which consisted of two key tasks: a meaning recall task and two pLDTs. The meaning recall task was identical to the meaning recall tasks presented during training. This task served as a measure of explicit knowledge pertaining to the novel words once all training tasks had been completed. Each novel word was presented once.

Following the meaning recall task, participants completed two pLDTs—one involving the recently learned novel words as primes and a second involving the familiar prime words. The order of these tasks was counterbalanced across participants.

Before the task commenced, participants were given instructions. Specifically, they would view two words in quick succession, and were asked to decide if the second (target) word was a real word in English or not. For half of the participants, the “A” key was pressed for a real word response and “L” for a non-word, whereas the key arrangement was reversed for the other half of participants. As per TG13, participants were also explicitly told that on some trials, the prime and target would be related.

**Figure 2.**
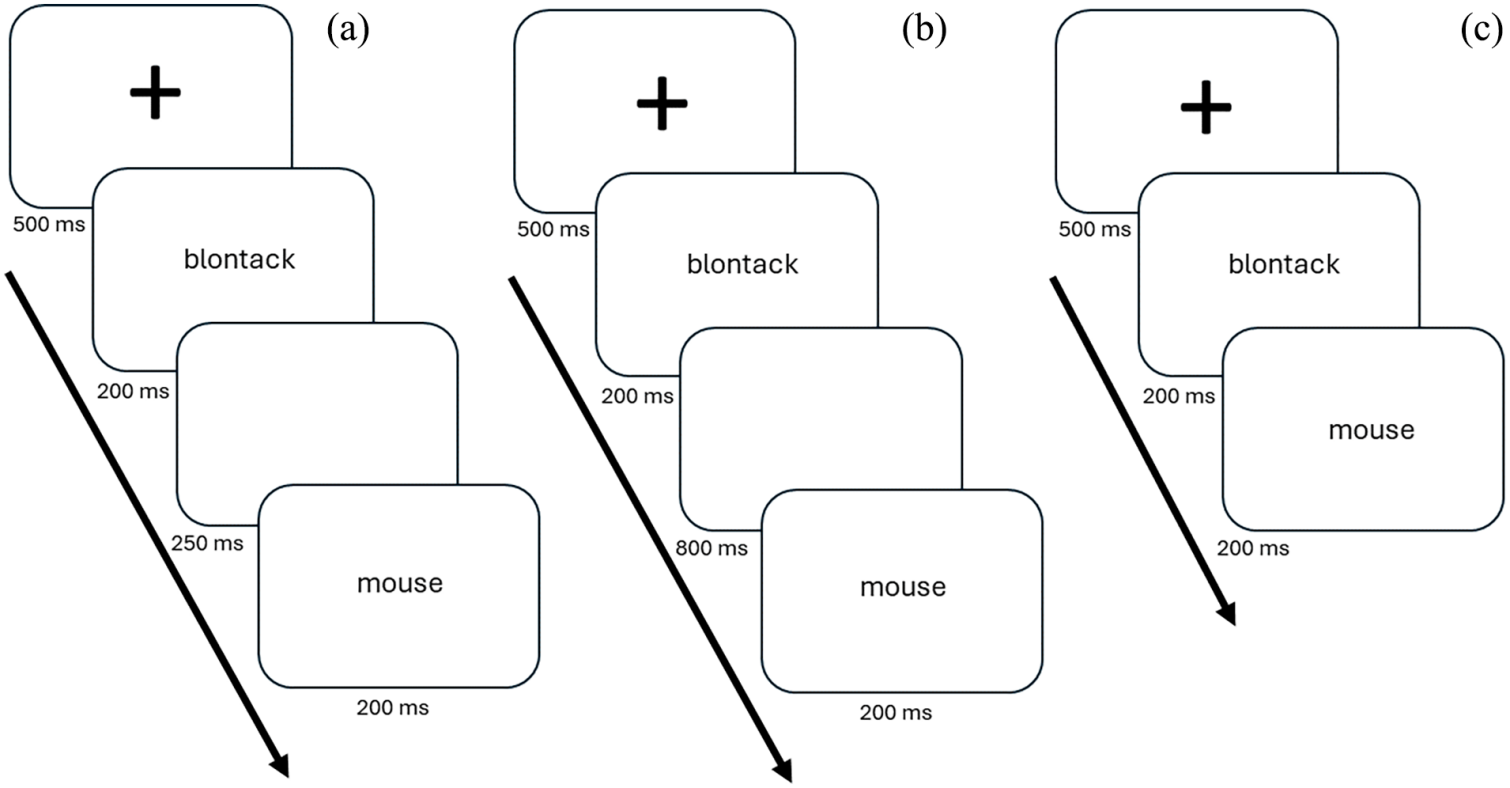
An illustration of the primed lexical decision tasks. Arrows represent the order of stimuli within a single trial. (a) Illustrates stimulus timings in Experiment 1; (b) illustrates stimulus timings in Experiment 2; (c) illustrates stimulus timings in Experiment 3. Notice that the only difference between Experiments 1 and 2 concerns the duration of the blank screen. In Experiment 3, the blank screen was removed to create a 200 ms SOA.

A single trial began with the presentation of a fixation cross for 500 ms. Then, the prime word appeared for 200 ms, followed by a blank screen for 250 ms (therefore creating an SOA of 450 ms). This was replaced by the target word which remained on screen for 200 ms. Participants could make their decision as soon as the target appeared and had up to 2,000 ms to respond (see Figure 2a). To encourage accurate and quick responses, feedback was provided in the form of a green tick for a correct response or a red cross for an incorrect response, along with the presentation of the response time for that trial, for 500 ms. This was then replaced by the fixation cross in preparation for the next trial. The presentation of trials was randomised across participants. However, the trial order was constrained so that there were no more than four consecutive trials of the same prime–target relatedness (related or unrelated), and no more than eight consecutive trials of the same target lexicality status (real or non-word). These constraints differ slightly from TG13 who allowed no more than three consecutive trials of the same prime–target relatedness and no more than four of the same target lexicality trials. This study also did not contend with constraining trial order based on time-of-testing (as did TG13), as words were not taught at different intervals (i.e., across different days). This meant that our novel lexical decision had half as many trials as the novel task in TG13, who taught participants 68 novel words across separate days.

Every target (real and non-word) was presented once per participant, with each prime appearing six times—on three occasions with a real word target and three occasions with a non-word. This meant that per participant, primes were not presented an equal number of times with a related and unrelated real word target. To counteract this, two versions of each pLDT were created, with participants completing just one version. For any given prime, it appeared twice with a related and once with an unrelated target in one version of the task, and twice with an unrelated and once with a related target in the other version. This meant that across participants, each prime was presented an equal number of times with a related and unrelated real word target.

The pLDTs were divided into three blocks. Each prime appeared twice per block, once with a real word target and once with a non-word target. Participants could use the time in between blocks to take a short break. Furthermore, the participants' cumulative accuracy rate—across blocks and tasks (novel and familiar)—was presented in between blocks, again to encourage accurate responses.

Each pLDT, therefore, consisted of 204 trials: 102 non-word target trials, 51 (word) related target trials and 51 (word) unrelated target trials. Accordingly, the relatedness-proportion was 0.5, and the non-word ratio was 0.67. In line with McNamara (2005), these parameters should at least permit the use of strategic priming mechanisms (expectancy generation and semantic matching, respectively), in conjunction with a sufficiently long SOA.

### Results

#### Explicit recall of novel word meaning

As per TG13, recall was considered as correct if the participant successfully recalled the core concept of the novel word’s meaning (e.g., “cat” in “is a type of cat that has stripes and is bluish-grey”). On average, participants successfully recalled 29/34 (84%; *SD* = ±0.19) novel word meanings, suggesting participants had acquired the meanings of the vast majority of the novel words. Indeed, 52/60 participants performed above chance level (68% of meanings recalled; *p* < .05 under a binomial distribution).^1^

#### Lexical decision times with familiar primes

Our analysis of data from the pLDTs followed the same procedures as TG13. Incorrect responses were removed, as were response times < 150 ms or > 1,500 ms that were considered to be outliers. Response time was used as the outcome variable in a linear mixed-effects model and was log-transformed to reduce the effect of positive skew on the data. Two separate models were constructed to separately analyse the data from the familiar and novel pLDTs, and included the fixed effect of prime–target relatedness (related or unrelated) as well as random intercepts for participants, primes, and targets. Random slopes were included if they significantly improved model fit. However, for both models in Experiment 1, no random slopes improved the fit of the model. We report Type-III tests of main effects to establish the effect of prime–target relatedness on lexical decision times.

Statistical models were run in *RStudio* (*R* version 4.0.4—R Core Team, 2022) using the *lmer* function from the *lme4* package (Bates et al., 2015). Estimated marginal mean (EMM) response times, reported in tables and figures, were calculated using the *emmeans* package (Lenth et al., 2019). Although response time was log-transformed when building statistical models, response time has been converted back to the response scale in tables and figures to aid interpretation.

**Figure 3.**
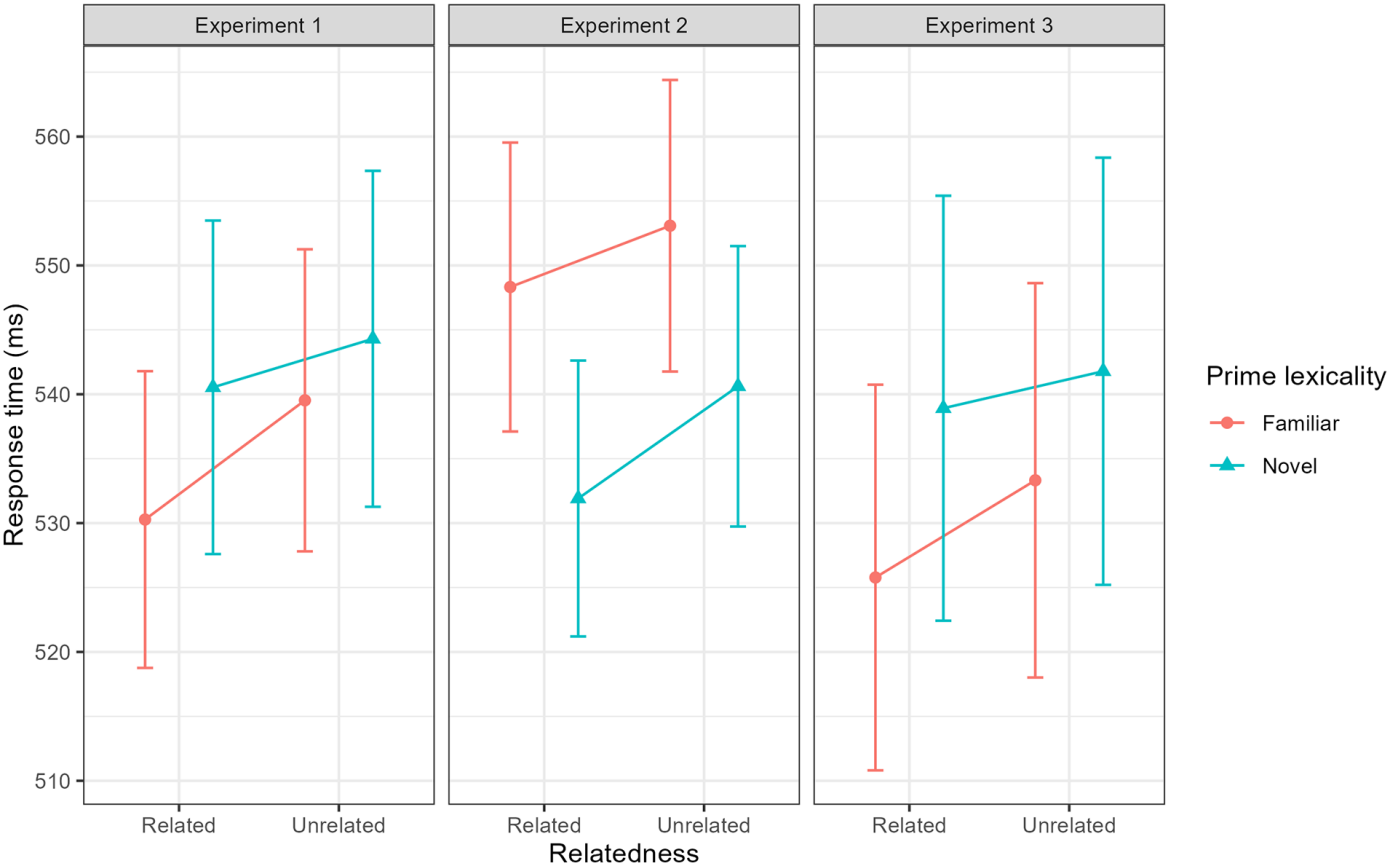
Semantic priming effects as a function of prime lexicality and experiment. Points represent estimated marginal mean response time and error bars represent standard error from the mean.

Due to a technical error, we removed data from one target in the familiar pLDT as this incorrectly appeared with two unrelated primes (across participants). This was the case for all analyses involving the familiar priming task reported throughout the article. In the familiar pLDT, there was a significant main effect of prime–target relatedness on lexical decision times (*F* (1, 5258.9) = 10.94, *p* < .001). Response time to the target was significantly faster following a related compared with an unrelated prime (see Table 1 and Figure 3), revealing a significant semantic priming effect.

**Table 1.** Estimated marginal mean response times in Experiment 1.

	Related	Unrelated	Priming effect (ms)
Familiar	530.28 (±11.52)	539.53 (±11.72)	9.25
Novel	540.54 (±12.94)	544.30 (±13.04)	3.76

Standard errors are presented in parentheses.

#### Lexical decision times with novel primes

The analysis revealed no significant main effect of prime–target relatedness on lexical decision times (*F* (1, 5224.1) = 1.90, *p* = .168). As can be seen in Table 1 and Figure 3, response times to the target were numerically quicker following a related prime; however, this did not reach significance.

#### Interim discussion—Experiment 1

The results of Experiment 1 replicate the method and findings of the “recent” condition of TG13’s first experiment—recently learned novel words, with an SOA of 450 ms between the prime and target in a pLDT, do not facilitate the recognition of associated (familiar) counterparts, whereas familiar words do.

As in TG13, the priming effect associated with the familiar primes was rather small. This is possibly due to the relatively weak prime–target associations on average (average forward association strength = .16). Given that each prime was presented three times throughout the experiment to provide a sufficiently large trial count, it is very difficult to identify three (relatively) strongly associated targets per prime (Tamminen & Gaskell, 2013). Furthermore, the backward association strength (BAS) scores in the familiar condition were even smaller (average BAS = .06). This may have limited the influence of semantic matching, which is most sensitive to the association between the target and prime (Neely & Keefe, 1989), weakening the overall priming effect. We return to this observation in the interim discussion of Experiment 2.

One noticeable difference between the findings of our experiment and TG13 is the overall increased response time in this experiment. We believe that one explanation for this concerns the participant sample. Our sample was older (mean age = 41 years) than that of TG13 (mean age = 21 years). Older participants have been shown to produce delayed lexical decision times (regardless of prime–target relatedness) compared with their younger counterparts (Gold et al., 2009; Madden, 1992), possibly due to general age-related changes in brain circuitry (Giorgio et al., 2010; though see Ramscar, 2022, for an alternative explanation for delayed lexical decisions in older individuals). Another possibility concerns the use of a web-based experiment, which has been found to elicit slower lexical decision latencies than face-to-face laboratory experiments (see Kim et al., 2021).

In Experiment 2, we increased the SOA from 450 to 1,000 ms. We believe that in doing so, the temporally limited hippocampal representations of the novel primes are provided with more time to engage before the presentation of the target. If prime meaning retrieval is complete, or enhanced relative to Experiment 1, before the presentation of the target, the effectiveness of strategic priming mechanisms (expectancy generation and/or semantic matching) should increase, possibly allowing an overall significant semantic priming effect to ensue (or at least produce a stronger effect than that found in Experiment 1).

## Experiment 2

### Methods

#### Participants

Participants were again recruited through *Prolific* and received £11 for their participation. In total, 68 participants completed the experiment, of which 60 contributed data to the analysis (*M* age = 41.51 years; *SD* = ±13.24; 27 males). The attrition breakdown for the eight rejected participants is as follows: exceeded the studies maximum completion time (*n* = 6), failure to provide a correct response in the priming task (*n* = 1); technical error (*n* = 1). All participants reported no known language-related disorders and English to be their native language. Potential participants could not access the experiment (on *Prolific*) if they took part in Experiment 1.

#### Stimuli, design, and procedure

The only methodological difference between Experiments 1 and 2 was an increase in SOA from 450 to 1,000 ms in the pLDTs. Specifically, the duration of the blank screen between the prime and target presentation was increased from 250 to 800 ms (see Figure 2b, above).

### Results

#### Explicit recall of novel word meaning

On average, participants successfully recalled 29/34 (86%; *SD* = ±0.19) of the novel word meanings, suggesting participants had acquired and retained the meanings of the vast majority of words. Indeed, 53/60 participants performed above chance level (range 3–100%). There was no significant difference in recall accuracy between experiments (*p* = .673).

#### Lexical decision times with familiar primes

The same data trimming and model fitting procedures as used in Experiment 1 were used again to analyse the lexical decision data collected in Experiment 2. We again report Type-III tests of main effects to explore the effect of prime–target relatedness on lexical decision times. As with Experiment 1, the random effects structure for both mixed-effects models in Experiment 2 included random intercepts for participants, primes and targets.

EMM response times for Experiment 2 are presented in Table 2. Unlike Experiment 1, there was no significant semantic priming effect in the familiar pLDT (*F* (1, 5304.9) = 2.55, *p* = .111). Nonetheless, there was a trend towards a significant effect of facilitated response time on related prime–target trials (see Table 2 and Figure 3).

**Table 2.** Estimated marginal mean response times in Experiment 2.

	Related	Unrelated	Priming effect (ms)
Familiar	548.32 (±11.21)	553.08 (±11.32)	4.76
Novel	531.91 (±10.71)	540.62 (±10.88)	8.71

Standard errors are presented in parentheses.

#### Lexical decision times with novel primes

There was a significant main effect of prime–target relatedness on response time in the novel pLDT (*F* (1, 5404.7) = 9.64, *p* = .002). Response time to the target was significantly quicker following a related compared with an unrelated prime (see Table 2 and Figure 3). Thus, there was a statistically significant semantic priming effect involving novel words without a consolidation period.

### Discussion of Experiment 2

The results of Experiment 2 show that recently learned novel words can facilitate the recognition of associated (familiar) counterparts. We suggest that these results could reflect one of both of two factors: 1) The recruitment of strategic priming mechanisms, and 2) activation of the newly encoded cortico-hippocampal representation, which regulate knowledge at this stage of learning. Crucially, both factors appear to necessitate a sufficiently long SOA.

An unexpected finding from Experiment 2 was that no significant priming effect was seen in the familiar condition. Given the presence of an effect in Experiment 1 this pattern requires some explanation. Why might we see a priming effect for these items at shorter SOA (in our Experiment 1 and TG13), but not at longer SOA? It seems plausible that the early effect of automatic spreading activation had faded before the presentation of the target, given the propensity for activation to soon dissipate following prime onset (Collins & Loftus, 1975). However, would we not expect to observe strategic priming for the familiar items too? A potential explanation as to why we might not relate to the BAS statistics that were discussed previously. As a reminder, the BAS statistics in the familiar condition are very low (average BAS of .06) and are considerably lower than the novel prime condition (average BAS of .16). There is evidence from prior work that semantic priming is more sensitive to BAS at long SOAs (compared with a shorter SOA). For example, Hutchison et al. (2008) found that the magnitude of semantic priming is predicted by BAS with an SOA of 1,200 ms (similarly, see Thomas et al., 2012 with an SOA of 800 ms). That is, weaker BAS is associated with weaker semantic priming.

BAS is believed to be associated with the strategic semantic priming mechanism of semantic matching—the participant checks back the association between the target and prime (Neely et al., 1989; Neely & Keefe, 1989). When an association is detected (from the target towards the prime), this can bias and facilitate the participant to respond with a word response in the pLDT (i.e., the target must be a word, as there is an association with the prime). However, when no association is detected but the target is a real word, the participant must override the bias to respond with non-word, inducing a slight delay in response time. The implication of this is that in this study, with very low BAS scores in the familiar condition, the ability of semantic matching to facilitate related target response time may have been rather minimal, as, overall, the association between the targets and their primes was very weak. In contrast, semantic matching may have had a greater impact in the novel condition where BAS statistics are considerably larger, and thus could have facilitated related target response time to a greater degree.

It is also important to acknowledge the delayed response times to familiar primes overall compared with the novel primes, as well as compared with the familiar primes of Experiment 1. One possible explanation for this is the same as for the lack of an overall priming effect. That is, early automatic effects of spreading activation should have dissipated before target onset given the long SOA. Similarly, if the effectiveness of the semantic matching strategy was impaired in the familiar prime condition, this should delay response times in both the related and unrelated prime–target conditions.

To summarise the results at this stage, novel words appear to require a sufficiently long SOA to engage in semantic priming, consistent with strategic priming mechanisms (McNamara, 2005) playing a role. This reliance on strategic mechanisms, we believe, is related to the state of underlying, neural representation. Specifically, consistent with the CLS account of lexical acquisition, novel word representations are initially supported by episodic memory, meaning they are unable to prime via automatic mechanisms such as spreading activation, which depends on integrated, cortical representations (Davis & Gaskell, 2009; TG13).

To further tease apart the role of automatic and strategic processes, we next sought to look at priming under circumstances in which there was a greater reliance on automatic processes. Although the relative engagement of strategic processing was likely weaker in Experiment 1 (450 ms) compared with Experiment 2 that used a longer SOA (1,000 ms), semantic priming in Experiment 1 was potentially influenced by strategic processing to some degree (McNamara, 2005; Neely, 1977). Hence, the absence of priming with novel words in Experiment 1 may reflect delayed or inefficient strategic processes (relative to familiar words), rather than an inefficiency of automatic priming mechanisms that would provide more direct support for an absence of cortical representation.

Considering this,^2^ we decided to run Experiment 3, which was identical to the previous experiments except that the SOA in the pLDTs was reduced to 200 ms. In line with McNamara (2005), semantic priming with a 200 ms SOA should be underpinned predominantly by automatic mechanisms, such as spreading activation.

## Experiment 3

### Methods

#### Participants

Participants were again recruited through *Prolific* and received £11 for their participation. In total, 62 participants completed the experiment, of which 60 contributed data to the analysis (*M* age = 37.13 years; *SD* = ±10.76; 31 males). One participant was excluded for failing to provide a correct response in the priming task (*n* = 1), whereas another participant encountered a technical error. All participants reported no known language-related disorders and English to be their native language. Potential participants could not access the experiment (on *Prolific*) if they took part in Experiment 1 or Experiment 2.

#### Stimuli, design, and procedure

The only methodology difference with respect to our previous two experiments concerned the SOA in the pLDTs. To achieve a 200 ms SOA, we simply removed the blank screen that interleaved prime and target presentation in Experiments 1 and 2 (see Figure 2).

### Results

#### Explicit recall of novel word meaning

On average, participants successfully recalled 26/34 (77%; *SD* = ±0.23) of the novel word meanings, with 44/60 participants performed above chance level (range 0–100%).

We compared explicit recall of meaning across experiments via a one-way between-subjects analysis of variance (ANOVA). Interestingly, the results revealed a significant main effect of experiment (*F* (3, 177) = 3.60, *p* = .029). Follow-up comparisons using the *emmeans* package in *RStudio* revealed significantly worse recall performance in Experiment 3 compared with Experiment 2 (*p* = .041), and there was a trend of lower recall in Experiment 3 relative to Experiment 1 (*p* = .074). There was no significant difference between Experiments 1 and 2 (*p* = .696). We did not expect to observe significantly worse recall in Experiment 3 as the training and recall procedures were identical across experiments. It was, therefore, prudent for us to investigate whether this could have affected the results related to the novel pLDT described below. Reassuringly, after removing participants who performed below chance level at meaning recall in Experiment 3 (*n* = 16), the lexical decision results were very similar to that comprising the full sample. Thus, although the Experiment 3 participants were generally worse at recalling novel word meanings, we do not believe that this had an adverse effect on performance in the pLDT.

#### Lexical decision times with familiar primes

The same data trimming and model fitting procedures as used in the two previous experiments were used to analyse the lexical decision data collected in Experiment 2. We again report Type-III tests of main effects to explore the effect of prime–target relatedness on lexical decision times.

For the mixed-effects model analysing lexical decision response times with familiar primes, the most parsimonious model included random intercepts for participants, primes and targets, as well as random slopes for the effect prime–target relatedness in relation to targets. EMM response times for Experiment 2 are presented in Table 3. Similar to Experiment 1 and unlike Experiment 2, there was a significant main effect of prime–target relatedness in the familiar pLDT (*F* (1, 419.74) = 6.05, *p* = .014), with significantly quicker response time following a related compared with an unrelated prime word (see Table 3 and Figure 3), revealing a significant semantic priming effect.

**Table 3.** Estimated marginal mean response times in Experiment 3.

	Related	Unrelated	Priming effect (ms)
Familiar	525.78 (±14.97)	533.36 (±15.31)	7.54
Novel	538.92 (±16.49)	541.79 (±16.58)	2.87

Standard errors are presented in parentheses.

#### Lexical decision times with novel primes

The most parsimonious model included random intercepts for participants and targets. There was no significant main effect of prime–target relatedness on response time in the novel pLDT (*F* (1, 4992.60) = 1.03, *p* = .311).

### Discussion of Experiment 3

Experiment 3 compared semantic priming between familiar and novel word primes under experimental conditions thought to bypass the effect of strategic processes. Although our two prior experiments have established a dependency of novel word priming on strategic priming mechanisms, the relatively long SOAs meant that we have yet to measure priming that is predominantly influenced by automatic mechanisms, such as spreading activation. We deemed this important to investigate, to probe the nature of cortical, semantic representation more directly.

Consistent with our predictions, we observed a significant semantic priming effect with familiar prime words, but not with recently learned novel words. We attribute this dissociation to differences in neural representations. Familiar prime words are likely to have established representations in semantic networks, meaning they can influence the processing of related concepts through mechanisms such as spreading activation (Collins & Loftus, 1975). Novel prime words, on the contrary, are believed to be represented in episodic memory rather than in cortical language networks, and therefore cannot influence related concepts through these same mechanisms (Davis & Gaskell, 2009; Tamminen & Gaskell, 2013).

We also found that delayed response time overall following novel relative to familiar primes. Similar to our interpretation above concerning relatedness effects, this could relate to a general absence of automatic processes in relation to new words, which is consistent with the view that offline consolidation periods, such as sleep, improve the automaticity in which lexical information is retrieved (McMurray et al., 2016).

## Exploratory analyses

The following sections present two pre-registered exploratory analyses.

### Analysis across experiments

As pre-registered, we performed an analysis in which we analysed the data collectively across experiments. The purpose here is to establish that any observed difference in outcomes is greater than one might expect from chance.

Through analysing the data collectively across experiments, we aimed to explicitly model the magnitude of semantic priming across prime lexicality and experiment/SOA length. “Experiment” was, therefore, included as a between-subjects factor in a linear mixed-effects model, along with “relatedness” and “lexicality” (both within-subjects). All factors were effect coded using the “contr.sum” function in R. As with our prior analyses, log-transformed response time was included as the outcome variable, and we report Type-III tests of main effects. Model estimates are provided below in Table 4.

**Table 4.** Predictors of response time across experiments.

Fixed effects	*F*	*p*
Relatedness	**22.86**	**<** **.001**
Lexicality	0.23	.630
Experiment	0.15	.861
Relatedness:Lexicality	1.15	.283
Relatedness:Experiment	0.18	.839
Lexicality:Experiment	**65.04**	**<** **.001**
Relatedness:Lexicality:Experiment	2.43	.088

Statistically significant terms are highlighted in bold. The model was configured over 32,137 observations, from 180 participants across 68 primes and 203 targets.

There was an effect of prime–target relatedness on response time, revealing an overall semantic priming effect (EMM-related trials = 535.73 ms, *SE* = ±7.18; unrelated trials = 541.46 ms, *SE* = ±7.26). The significant interaction between lexicality and experiment was driven by significantly slower overall response time following a familiar compared with a novel prime in Experiment 2 (EMM familiar primes = 551.73 ms, *SE* = ±12.69; novel primes = 536.43 ms, *SE* = ±12.33; *p* = .001). In Experiment 3, trials involving familiar primes were responded to more quickly than novel primes (EMM familiar primes = 527.04 ms, *SE* = ±12.13; novel primes = 545.39 ms, *SE* = ±12.55; *p* < .001).

Finally, there was a trend of a significant three-way interaction between all three factors *(p* = .088). This was explored further by comparing response time between related and unrelated prime–target pairings, separately for novel and familiar primes in each experiment. This resulted in six contrasts (1: *familiar related vs unrelated Experiment 1*; 2: *novel related vs unrelated Experiment 1*; 3: *familiar related vs unrelated Experiment 2*; 4: *novel related vs unrelated Experiment 2*; 5: *familiar related vs unrelated Experiment 3*; 6: *novel related vs unrelated Experiment 3*), with a Holm-Bonferroni *p*-value adjustment applied to control for multiple comparisons. In Experiment 1, there was a significant semantic priming effect involving familiar (*p* = .003) but not novel (*p* = .689) primes. In Experiment 2, there was a significant semantic priming effect involving novel (*p* = .015) but not familiar (*p* = .449) primes. Finally, in Experiment 3, there was a trend of a significant semantic priming effect involving familiar (*p* = .107) but not novel (*p* = .689). These contrasts, therefore, are consistent with the results reported in our main analyses.

### Removal of participants based on performance

The models reported throughout this article were configured over a sample of 60 participants, who contribute at least one data point to the analysis. However, it is possible that some participants performed relatively poorly in the priming tasks, which might have had an effect on the results. To determine any detrimental effect that such participants could have had on the observed results, we performed a follow-up analysis whereby we excluded participants who performed below chance level at classifying word/non-word targets. Chance level performance was determined as 63%, which was calculated by performing 10,000 simulations of 51 Bernoulli trials (51 being the number of trials per relatedness condition in the pLDT). Comparing correct responses with a critical alpha level of .05 revealed that ≥ 32 correct trials (or 63%) corresponded to above chance level of performance.

Six models were thus configured, reanalysing the familiar/novel pLDTs in each experiment with below chance performers removed. Type-III main effects of prime–target relatedness are reported as per the main analysis, and the models are summarised in Table 5.

**Table 5.** Model summaries with below chance performers removed.

Experiment	Prime lexicality	*n* participants removed	*F*-value	*p*-value
Experiment 1 (450 ms SOA)	Familiar primes	7	9.11	.003
	Novel primes	7	0.18	.672
Experiment 2 (1,000 ms SOA)	Familiar primes	6	1.71	.191
	Novel primes	6	7.29	.007
Experiment 3 (200 ms SOA)	Familiar primes	9	9.87	.002
	Novel primes	9	0.76	.385

SOA: stimulus onset asynchrony.

The results of this follow-up analyses suggest that the potential influence of poor performance in the pLDTs was minimal. That is, after removing participants who performed below chance level, the significance of our terms did not change compared with the main analysis. In Experiment 1, seven participants were identified as performing below chance level. With these participants removed, priming continued to be observed for the familiar but not for the novel primes. Likewise, six participants were identified as performing below chance level in Experiment 2. Priming continued to be observed for the novel but not for the familiar primes following the removal of these participants’ data. Finally, nine participants performed below chance level in Experiment 3, and after removing these data, priming continued to be observed with familiar but not with novel primes.

## General discussion

This series of studies aimed to investigate the representation of recently learned words and specifically how they might interact with representations of known words via strategic processes. It is argued that new words are initially represented episodically (Davis & Gaskell, 2009), and that this explains why they do not prime semantically related known words in the same way that other known words do. An issue for this hypothesis, therefore, is reports of novel word semantic priming in some studies (Bakker et al., 2015; Balass et al., 2010; Perfetti et al., 2005). We propose that such novel word priming is dependent on strategic processing, compared with known words that may additionally prime under more automatic conditions, thus implicating representational differences across word types. We tested this across three experiments—Experiment 1 recruited a 450 ms long SOA, Experiment 2 recruited a 1,000 ms SOA, while Experiment 3 recruited a 200 ms SOA. Because strategic mechanisms depend on sufficiently long SOAs (McNamara, 2005; Neely, 1977), we predicted to only observe significant semantic priming with novel words in Experiment 2. Consistent with these predictions, novel words did not prime existing words in Experiments 1 and 3 but did so in Experiment 2. This pattern of priming was also supported by an exploratory analysis, which analysed the data collectively across experiments.

The idea that new words may rely more heavily on controlled and strategic semantic processes has been proposed previously (Bakker et al., 2015). We believe that this study provides novel evidence for this proposal by showing that novel words require a relatively long SOA to engage in semantic priming. Under shorter SOA conditions (i.e., 200 ms), where the effectiveness of strategic processing is presumably more limited, semantic priming may be attributed more heavily towards automatic priming mechanisms such as spreading activation, which possibly depends on integrated semantic representations (Tamminen & Gaskell, 2013). This can, therefore, explain why familiar, but not novel, prime words were capable of priming in Experiment 3, given that familiar words are likely to have well-established semantic representations in cortical language networks.

This pattern of results compliments other work suggesting a significant interplay between novel and familiar words that is qualitatively distinct from the interplay between familiar words. For instance, in recent years, research has shown that recently learned words can compete with phonologically related words (Kapnoula & McMurray, 2016; Kapnoula et al., 2015; Weighall et al., 2017), compared with earlier work suggesting that consolidation periods are required for competition effects to emerge (Dumay & Gaskell, 2007; Dumay et al., 2004; Gaskell & Dumay, 2003). Critically, the presence of competition effects appears to be at least partly dependent on the nature of the task measuring competition. That is, studies which report significant effects recruited a visual world paradigm (VWP), in which participants are encouraged to fixate on a particular on-screen object based on incoming speech. Competition effects are found when the target object (e.g., biscuit) is paired with a phonological competitor (e.g., beetle), reflecting the co-activation of phonologically similar lexical representations. Studies which report non-significant effects, however, used a pause-detection task (Mattys & Clark, 2002), where participants are instructed to make a speeded decision regarding the presence of a pause inserted within an audible word (e.g., *cathedr_al*).

Weighall et al. (2017) provided an account to explain these discrepancies by considering how episodic, hippocampal representations may contribute differently across these tasks, by considering the relative speed of processing between new and familiar words. That is, according to the CLS account, novel words are processed more slowly than familiar words because the mediating hippocampal pathway of new words provides only an indirect route of lexical knowledge that is activated with a lower priority compared with integrated cortical representations of familiar words (Davis & Gaskell, 2009; Lindsay & Gaskell, 2010). In terms of the pause-detection task, participants are instructed to make a swift, speeded decision, which may be too quick for the hippocampal pathway to engage sufficiently to influence behaviour. The VWP, on the contrary, provides a more continuous measure of competition along an extended time course, which could “be better able to incorporate information arriving relatively slowly via recently learned hippocampal links” (Weighall et al., 2017, p.24). It is interesting to consider this proposition in the context of our findings. Speculatively, the 450 ms SOA of Experiment 1 could have been too short for new words and their meanings to be retrieved sufficiently in time before the presentation of the target. In turn, this would limit the effectiveness of any strategic mechanism on semantic priming. In contrast, the 1,000 ms SOA of Experiment 2 could have provided a sufficiently long temporal window for novel word retrieval, allowing these meanings to be used in conjunction with strategic mechanisms. Future work involving neuroimaging techniques is nonetheless required to investigate these claims around processing speed more objectively.

There is now a considerable amount of work showing that new words can influence how other existing words are processed (McMurray et al., 2016). Generally speaking, tasks that promote more automatic modes of lexical/semantic access, such as a semantic priming task with a relatively short SOA (Coutanche & Thompson-Schill, 2014; Tamminen & Gaskell, 2013; van der Ven et al., 2015, 2017) or a pause-detection task measuring competition between phonological similar words (Dumay et al., 2004; Dumay & Gaskell, 2007; Gaskell & Dumay, 2003) are associated with non-significant effects. However, if the task promotes more strategic processing, such as a semantic priming task with a relatively long SOA (including Experiment 2 of this study and that of Balass et al., 2010; Perfetti et al., 2005) or a VWP measuring competition effects (Kapnoula & McMurray, 2016; Kapnoula et al., 2015; Weighall et al., 2017), then significant effects appear to emerge. This is consistent with the notion that newly acquired skills and behaviour are generally executed with input from attentional systems (Chein & Schneider, 2012). Thus, the interactive capabilities of recently learned novel words are perhaps not best conceived as an “all or nothing” phenomenon (Walker et al., 2019). Rather, these effects may be best viewed along a continuum that is dependent on not only stages of sleep-related consolidation, but also the amount of processing automaticity that is required by the task (as well as individual differences in higher-order functioning).

In conclusion, this study has demonstrated that recently learned novel words have the capability to semantically prime existing words. However, it would seem that such effect may be sourced from strategic, compared with automatic, priming mechanisms shortly after learning, compared with known words that may semantically prime under automatic conditions. This finding provides support for the idea that new words are represented in a qualitatively different way to known, familiar words.

## Supplemental Material

sj-docx-1-qjp-10.1177_17470218241306747 – Supplemental material for Revisiting novel word semantic priming: The role of strategic priming mechanisms Supplemental material, sj-docx-1-qjp-10.1177_17470218241306747 for Revisiting novel word semantic priming: The role of strategic priming mechanisms by Lewis V Ball, Perrine Brusini and Colin Bannard in Quarterly Journal of Experimental Psychology

## Notes

1. For all three experiments, we repeated the novel primes lexical decision time analyses with participants who performed below chance level in the meaning recall task removed. The results were very similar to those reported in the article, with the same direction and significance of effects.

2. We also thank an anonymous reviewer who motivated this experiment.
