# Supplementary material for: Revisiting novel word semantic priming: The role of strategic priming mechanisms
Source: Q J Exp Psychol (Hove). 2024 Dec 21;78(10):2284–98. doi: 10.1177/17470218241306747 (PMC12432285; doi:10.1177/17470218241306747)
Supplement: sj-docx-1-qjp-10.1177_17470218241306747 – Supplemental material for Revisiting novel word semantic priming: The role of strategic priming mechanisms [file sj-docx-1-qjp-10.1177_17470218241306747.docx]

Supplementary Material for:

Revisiting Novel Word Semantic Priming: The Role of Strategic Priming Mechanisms

Lewis. V. Ball^a,b^, Perrine Brusini^a^, Colin Bannard^c^

*^a^ Department of Psychology, University of Liverpool, Liverpool, L69 7ZA, United Kingdom*

*^b^ Department of Psychology, University of York, York, YO10 5DD, United Kingdom*

*^c^ Department of Linguistics and English Language, University of Manchester, Manchester, M13, 9PL, United Kingdom*

Corresponding author: Lewis Ball ([lewis.v.ball@gmail.com](mailto:lewis.v.ball@gmail.com))

Data availability statement: Trial-level data and analysis code are available on the Open Science Framework: [https://osf.io/6xvzp/](https://osf.io/6xvzp/?view_only=49c7df9f94a946ae8583bd2c3ce6982c). Study materials can be found in the appendices of this document.

Supplementary Material A

Table A.1: Novel words, their meanings, their related real word targets and nonwords derived from the real word targets that were used in the novel lexical decision task.

| Word | Meaning | Real word target 1 | Real word target 2 | Real word target 3 | Nonword target 1 | Nonword target 2 | Nonword target 3 |
| --- | --- | --- | --- | --- | --- | --- | --- |
| agglem | is a type of baby that is premature and underweight | child | cry | infant | chyld | cro | Inlant |
| ardoff | is a type of beef that is British and comes from calves | steak | meat | roast | steat | veat | oast |
| blontack | is a type of cat that has stripes and is blue-ish grey | dog | mouse | kitten | dox | wouse | kitgen |
| chebbor | is a type of skirt that is flowery and made of silk | dress | blouse | shirt | driss | blousa | shirf |
| chisdow | is a type of prison that is for murderers and is located in the U.S. | jail | bar | cell | jais | ber | rell |
| dawtatt | is a type of neck that is short and freckled | shoulder | throat | tie | shounder | throad | kie |
| dobbir | is a type of knife that is often used by butchers and is very sharp | fork | cut | blade | fosk | vut | blada |
| entelem | is a type of cream that is organic and low in fat | whip | coffee | cheese | whis | coftee | cheete |
| eritriff | is a type of meadow that buffalo graze in and that was created by Native Americans | field | grass | flower | fielm | prass | flowen |
| feckton | is a type of knight that carries a banner and protects the helpless | armor | soldier | sword | arhor | solpier | swort |
| flimmir | is a type of sheep that lives in Scotland and has soft hair | wool | lamb | herd | woot | pamb | hird |
| gahoon | is a type of candle that has a fragrance and has an especially bright flame | light | wax | flame | jight | wex | flome |
| glain | is a type of leg that is long and very muscly | arm | body | walk | arn | sody | walp |
| heprit | is a type of face that has had plastic surgery and looks completely different | eyes | nose | smile | oyes | nosa | smige |
| hoddar | is a type of ring that is silver and engraved | finger | wedding | diamond | cinger | wodding | diawond |
| jabbary | is a type of fog that happens in equatorial areas and appears very quickly | mist | smog | thick | misp | swog | theck |
| kerple | is a type of pan that is battery-heated and used for camping | pot | cook | fry | pog | wook | bry |
| konrith | is a type of maid that comes in once a day and takes care of pets | clean | servant | butler | cleah | sermant | bunler |
| loodit | is a type of pistol that carries 20 bullets and can fire very quickly | gun | shoot | rifle | gug | shoog | rikle |
| lupitat | is a type of lemon that is seedless and imported from Mexico | lime | sour | orange | limi | rour | orenge |
| meckalen | is a type of fist made with the thumb on top and a bent wrist | fight | hand | punch | feght | hond | ponch |
| merdut | is a type of bread that is dark brown and has nuts in it | butter | dough | loaf | vutter | mough | loat |
| ospont | is a type of path that is paved and occurs in parks | road | trail | way | roat | truil | woy |
| peckolet | is a type of drawing that is a portrait and is in neon colours | art | picture | sketch | ast | pictere | skitch |
| poffren | is a type of shoe that has a strap and is made of plastic | foot | sock | lace | foet | seck | labe |
| quammish | is a type of book that has pictures and is oversize | read | school | study | pead | schood | stidy |
| quemmer | is a type of tooth that is weak and is discoloured | decay | ache | brush | debay | uche | bresh |
| slethy | is a type of ear that belongs to a mammal and is folded | hear | sound | head | vear | soind | heax |
| speth | is a type of cow that has a hairy tail and has giant horns | milk | calf | bull | rilk | calt | jull |
| tobbin | is a type of mirror that is circular and is convex | reflection | image | glass | seflection | umage | gless |
| uvar | is a type of monk that lives in Tibet and fasts for seven days at a time | priest | monastery | religion | proest | modastery | teligion |
| vilchy | is a type of pill that lowers cholesterol and blood pressure | medicine | drug | aspirin | tedicine | drig | asmirin |
| vorent | is a type of needle that is made of platinum and can make very small | thread | sew | pin | threal | gew | pid |
| waba | is a type of crown that is worn by monarchs and is made of rubies | king | jewel | queen | fing | jefel | queel |

Table A.2: Familiar prime words, their related, real word targets and nonwords derived from the real word targets that were used in the familiar lexical decision task.

| Word | Real word target 1 | Real word target 2 | Real word target 3 | Nonword target 1 | Nonword target 2 | Nonword target 3 |
| --- | --- | --- | --- | --- | --- | --- |
| ambulance | emergency | siren | accident | emermency | giren | accicent |
| balloon | air | helium | float | oir | hesium | fload |
| binder | folder | notebook | paper | volder | notegook | waper |
| bruise | hurt | pain | hit | hurp | pait | hib |
| burglar | thief | robber | steal | thiel | tobber | steab |
| cannon | ball | fire | weapon | byll | fite | weanon |
| circus | clown | animal | carnival | clewn | animad | carpival |
| clinic | doctor | sick | health | hoctor | bick | heamth |
| coffin | dead | burial | grave | sead | butial | frave |
| dart | board | game | throw | boarf | gamu | thriw |
| eraser | pencil | mistake | rubber | pencid | misvake | subber |
| flask | wine | bottle | whiskey | wina | bittle | whilkey |
| flour | cake | bake | sugar | cace | dake | sutar |
| frog | toad | hop | jump | toak | kop | fump |
| herb | spice | tea | garden | spoce | toa | larden |
| ketchup | mustard | red | tomato | muskard | rer | togato |
| lizard | reptile | snake | green | reppile | sneke | dreen |
| medal | gold | award | honor | golp | awarn | hosor |
| nun | convent | church | sister | lonvent | chorch | tister |
| oyster | clam | shell | pearl | claf | shull | pearn |
| paddle | row | oar | canoe | rop | oad | casoe |
| parcel | package | post | box | dackage | posk | jox |
| pebble | rock | stone | beach | vock | stine | neach |
| raisin | grape | prune | fruit | grare | prane | frait |
| salad | lettuce | dressing | bowl | lettace | bressing | bewl |
| sausage | breakfast | pork | bacon | breamfast | porl | gacon |
| slug | worm | slow | snail | worb | sfow | snoil |
| termite | bug | wood | pest | byg | bood | mest |
| tiger | lion | jungle | stripe | liot | dungle | strepe |
| towel | cloth | wet | wash | clath | det | wosh |
| vampire | blood | bat | fangs | bloot | baf | nangs |
| vinegar | oil | bitter | salt | oid | vitter | sall |
| wallet | money | purse | leather | momey | pursa | leathen |
| wasp | sting | bee | nest | stong | kee | nesk |


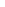


Supplementary Material B

Table B.1: Sentences used in the sentence plausibility task of the training phase for each novel word.

| Novel word | Sentence | Plausibility |
| --- | --- | --- |
| Agglem | The doctor was happy to announce the survival of the agglem. | Plausible |
|  | The midwife carefully picked up the agglem. | Plausible |
|  | The doctor was astounded by the growth of the agglem. | Plausible |
|  | The train was packed with commuters on their way to agglem. | Implausible |
| Ardoff | The man didn't care for vegetarian food so he chose a burger with ardoff. | Plausible |
|  | The experienced chef was very helpful and recommended. the ardoff. | Plausible |
|  | The guest examined the menu and was torn between the chicken and ardoff. | Plausible |
|  | The child's favourite game was ardoff. | Implausible |
| Blontack | The woman liked to listen to the purring of her blontack. | Plausible |
|  | The vet was pleased by the recovery of the blontack. | Plausible |
|  | The woman was woken by the paws of her hungry blontack. | Plausible |
|  | The monkey was too frightened to climb the blontack. | Implausible |
| Chebbor | The fashion designer was pleased with the design of the new chebbor. | Plausible |
|  | The woman went to the party wearing her new chebbor. | Plausible |
|  | The mannequin was wearing the new chebbor. | Plausible |
|  | The man's car broke down and was taken to the chebbor. | Implausible |
| Chisdow | The judge sentenced the criminal to two years in chisdow. | Plausible |
|  | The guard protected the tall walls of the chisdow. | Plausible |
|  | The criminals prepared a plan to escape from the chisdow. | Plausible |
|  | The book was written in 18-century chisdow. | Implausible |
| Dawtatt | The man found the shirt otherwise comfortable but the collar was too tight around his dawtatt. | Plausible |
|  | The fast car put a lot of strain on the driver's dawtatt. | Plausible |
|  | The man wore a scarf around his dawtatt. | Plausible |
|  | The paramedic rushed to the scene of the dawtatt. | Implausible |
| Dobbir | The cook sliced the lamb with his dobbir. | Plausible |
|  | The man sliced his finger on the dobbir. | Plausible |
|  | The cutlery drawer had only one dobbir. | Plausible |
|  | The boat alerted the coast guard when it began to take on dobbir. | Implausible |
| Entelem | The child asked the waiter for cookies and entelem. | Plausible |
|  | The baker was pleased by the taste of the entelem. | Plausible |
|  | The chef served the trifle with entelem. | Plausible |
|  | The student erased their work using their entelem. | Implausible |
| Eritriff | The children ran out and rolled in the dewy eritriff. | Plausible |
|  | The sun quickly set over the green plains of the eritriff. | Plausible |
|  | The horse ran happily through the eritriff. | Plausible |
|  | The politician delivered a strong and powerful eritriff. | Implausible |
| Feckton | The maiden locked in the tower was rescued by a handsome feckton. | Plausible |
|  | The medieval banquet was attended by the brave feckton. | Plausible |
|  | The village was saved thanks to the heroics of the feckton. | Plausible |
|  | The accountant was shocked after reading the latest figures in the feckton. | Implausible |
| Flimmir | The owner of the farm was horrified when she saw in the field only one flimmir. | Plausible |
|  | The farmer set out to round up of all the flimmir. | Plausible |
|  | The farmer began shearing the flimmir. | Plausible |
|  | The volcano erupted and caused disruption on the flimmir. | Implausible |
| Gahoon | The man was mindful of fire safety and put out the gahoon. | Plausible |
|  | The restaurant prepared for dinner by lighting the gahoon. | Plausible |
|  | The room had a pleasant smell from the fumes of the gahoon. | Plausible |
|  | The students danced together at the gahoon. | Implausible |
| Glain | The athlete couldn't run after breaking his glain. | Plausible |
|  | The personal trainer explained that squating helps train muscles in the glain. | Plausible |
|  | The rugby player went to the gym to train her glain. | Plausible |
|  | The printer required attention after it ran out of glain. | Implausible |
| Heprit | The man felt confident for the first time because of his heprit. | Plausible |
|  | The ball struck the person's heprit. | Plausible |
|  | The surgeon was pleased with the result of the heprit. | Plausible |
|  | The receptionist blew their nose into a heprit. | Implausible |
| Hoddar | The man asked her to marry him and gave her an expensive hoddar. | Plausible |
|  | The diver took no risks and removed their expensive hoddar. | Plausible |
|  | The marriage ceremony finished after the bride and groom each received their hoddar. | Plausible |
|  | The driver explained that the journey will take longer due to the closure of the hoddar. | Implausible |
| Jabbary | The plane could not land due to a heavy jabbary. | Plausible |
|  | The driver had trouble seeing through the jabbary. | Plausible |
|  | The referee cancelled the game as they could not see the other end of the pitch due to the jabbary. | Plausible |
|  | The priest was pleased by the attendence at this morning's jabbary. | Implausible |
| Kerple | The wife made an omelette on her non-stick kerple. | Plausible |
|  | The child burnt their hand on the hot kerple. | Plausible |
|  | The ingreditents were placed into the kerple. | Plausible |
|  | The zoo began to release animals back into the kerple. | Implausible |
| Konrith | The man didn't have time to take care of his guinea pigs so he hired a professional konrith. | Plausible |
|  | The woman's dog was fed by the konrith. | Plausible |
|  | The cat was looked after by the konrith. | Plausible |
|  | The man broke the computer after spilling his konrith. | Implausible |
| Loodit | The sheriff threatened the highwayman with his loodit. | Plausible |
|  | The silence was quickly broken by the shooting of the loodit. | Plausible |
|  | The race commenced after the firing of the loodit. | Plausible |
|  | The athlete was nervous at the prospect of competing in the loodit. | Implausible |
| Lupitat | The man preferred his iced tea with a fresh slice of lupitat. | Plausible |
|  | The chef squeezed the juicy lupitat. | Plausible |
|  | The baby pulled a disgusted face after biting into the lupitat. | Plausible |
|  | The opera singer was forced to sing louder when they broke their lupitat. | Implausible |
| Meckalen | The man was furious and hit the table with his meckalen. | Plausible |
|  | The patient was asked to open and close their meckalen. | Plausible |
|  | The boxer clenched their meckalen. | Plausible |
|  | The guest complained to the manager after finding a hair in their meckalen. | Implausible |
| Merdut | The woman living next to a bakery loved the smell of fresh merdut. | Plausible |
|  | The jam was spread over the merdut. | Plausible |
|  | The man made a sandwich with the merdut. | Plausible |
|  | The student went to the library to return a merdut. | Implausible |
| Ospont | The old man got lost after following the wrong ospont. | Plausible |
|  | The engineers installed floodlights to light up the ospont. | Plausible |
|  | The runners ran along the ospont. | Plausible |
|  | The house was placed on the market for a cheap ospont. | Implausible |
| Peckolet | The parents were impressed when the child painted a lovely peckolet. | Plausible |
|  | The artist made a lot of money after selling their peckolet. | Plausible |
|  | The man bought pencils and pens to begin their peckolet. | Plausible |
|  | The woman sold her phone to the peckolet. | Implausible |
| Poffren | The woman broke one of her heels and needed to buy a new poffren. | Plausible |
|  | The friends queued up all night for a chance to buy the latest poffren. | Plausible |
|  | The girl ran to school wearing her new poffren. | Plausible |
|  | The computer began to overheat and caused a poffren. | Implausible |
| Quammish | The librarian could not find the quammish. | Plausible |
|  | The blurb explained the contents of the quammish. | Plausible |
|  | The author began writing their new quammish. | Plausible |
|  | The cafe was busy thanks to its tasty quammish. | Implausible |
| Quemmer | The dentist pulled out the patient's quemmer. | Plausible |
|  | The woman took painkillers to ease the pain of her quemmer. | Plausible |
|  | The woman took great care to clean her quemmer. | Plausible |
|  | The dusty track descends to a quemmer. | Implausible |
| Slethy | The doctor told the old lady the loud music had damaged the drum of her cat's slethy. | Plausible |
|  | The rabbit had an ache in their slethy. | Plausible |
|  | The music quickly entered the DJ's slethy. | Plausible |
|  | The man went into the kitchen to wash the slethy. | Implausible |
| Speth | The vet inspected the hooves of the speth. | Plausible |
|  | The car stopped when crossing the road was a black and white speth. | Plausible |
|  | The farmer had a great affection for their speth. | Plausible |
|  | The bartender poured the guest a pint of speth. | Implausible |
| Tobbin | The girl enjoyed watching herself in the tobbin. | Plausible |
|  | The child accidently pushed over and cracked the tobbin. | Plausible |
|  | The wall was covered by a large tobbin. | Plausible |
|  | The woman took her dog for a walk in the tobbin. | Implausible |
| Uvar | The man enjoyed meditating so much that he became a deeply religious uvar. | Plausible |
|  | The church procession was led by the uvar. | Plausible |
|  | The religious person decided to become an uvar. | Plausible |
|  | The professor demonstrated the laws of physics using his uvar. | Implausible |
| Vilchy | The patient needed a glass of water to swallow the vilchy. | Plausible |
|  | The doctor prescribed the patient a vilchy. | Plausible |
|  | The man's knee pain improved after swallowing a vilchy. | Plausible |
|  | The teacher was quick to find fault in the student's vilchy. | Implausible |
| Vorent | The man fixed a hole in his child's clothing with the vorent. | Plausible |
|  | The child accidently pricked their finger with the vorent. | Plausible |
|  | The sewing class began with a demonstration of how to use a vorent. | Plausible |
|  | The mechanic shook hands with the vorent. | Implausible |
| Waba | The princess hoped that one day she could carry on her head the waba. | Plausible |
|  | The burglars sneaked into the palace with the intention of stealing the waba. | Plausible |
|  | The museum unveiled the new 16th century waba. | Plausible |
|  | The women typed on her waba. | Implausible |
